# Strengthening human genetics research in Africa: report of the 9th meeting of the African Society of Human Genetics in Dakar in May 2016

**DOI:** 10.1017/gheg.2017.3

**Published:** 2017-08-04

**Authors:** R. Ndiaye Diallo, M. Gadji, B. J. Hennig, M. V. Guèye, A. Gaye, J. P. D. Diop, M. Sylla Niang, P. Lopez Sall, P. M. Guèye, A. Dem, O. Faye, A. Dieye, A. Cisse, M. Sembene, S. Ka, N. Diop, S. M. Williams, E. Matovu, R.S. Ramesar, A. Wonkam, M. Newport, C. Rotimi, M. Ramsay

**Affiliations:** 1 Cheikh Anta Diop University of Dakar (UCAD), Dakar, Senegal; 2 London School of Hygiene & Tropical Medicine, London, UK; 3 National Human Genome Research Institute, National Institutes of Health, Bethesda, Maryland, USA; 4 Department of Epidemiology and Biostatistics, Institute of Computational Biology, Case Western Reserve University, Cleveland, USA; 5 Faculty of Veterinary Medicine, Makerere University, Kampala, Uganda; 6 Division of Human Genetics, Department of Pathology, Institute of Infectious Diseases and Molecular Medicine, Faculty of health Sciences, University of Cape Town, Cape Town, South Africa; 7 Brighton and Sussex Medical School (BSMS), Farmer, Brighton, UK; 8 Sydney Brenner Institute for Molecular Bioscience and Division of Human Genetics, Faculty of Health Sciences, University of the Witwatersrand, Johannesburg, South Africa

**Keywords:** African Genetic Research, African Society of Human Genetics, Dakar, Meeting report

## Abstract

The 9th meeting of the African Society of Human Genetics, in partnership with the Senegalese Cancer Research and Study Group and the Human Heredity and Health in Africa (H3Africa) Consortium, was held in Dakar, Senegal. The theme was *Strengthening Human Genetics Research in Africa.* The 210 delegates came from 21 African countries and from France, Switzerland, UK, UAE, Canada and the USA. The goal was to highlight genetic and genomic science across the African continent with the ultimate goal of improving the health of Africans and those across the globe, and to promote the careers of young African scientists in the field. A session on the sustainability of genomic research in Africa brought to light innovative and practical approaches to supporting research in resource-limited settings and the importance of promoting genetics in academic, research funding, governmental and private sectors. This meeting led to the formation of the Senegalese Society for Human Genetics.

## Abstract (fr)

Le 9^ème^ congrès de la Société Africaine de Génétique Humaine, en partenariat avec le Groupe d’Etude et de Recherche sur le Cancer (GERC) et le Consortium H3Africa, s’est tenu à Dakar, au Sénégal. Le thème était «*Renforcer la recherche en Génétique Humaine en Afrique*». Les 210 participants sont venus de 21 pays africains et de six non africains. L’objectif était de valoriser la génétique et la génomique à travers l’Afrique avec comme but ultime d’améliorer la santé des populations, et de promouvoir les carrières des jeunes chercheurs Africains. Une session sur la pérennité de la recherche génomique a révélé des approches innovantes et pratiques supportant la recherche dans des contextes de ressources limitées et l’importance de promouvoir la formation universitaire en génétique, le financement de la recherche par les gouvernements et le privé. Ce congrès conduisit à la création de la Société Sénégalaise de Génétique Humaine.

## Introduction

The 9th meeting of the African Society of Human Genetics (AfSHG) was hosted in Dakar, Senegal, from 15 to 17 May 2016 as a joint conference with the Senegalese Cancer Research and Study Group (GERC) and the Human Heredity and Health in Africa (H3Africa) Consortium to discuss and explore mutual challenges and significant synergies in genomic science across the African continent and globally. The theme of the meeting was ‘*Strengthening Human Genetics Research in Africa*’ and it attracted a total of 210 delegates representing 27 nationalities including Senegal, Mali, South Africa, Congo, Erithrea, Democratic Republic of Congo, Benin, Cameroon, Burkina Faso, Zimbabwe, Morocco, Ethiopia, Nigeria, Uganda, Tanzania, Sudan, Tunisia, Rwanda, Ghana, Egypt, Botswana and six non-African countries. This was the first time the AfSHG meeting was held in an exclusively francophone country, a choice motivated by the desire for a closer bond with African scientists from 13 African countries whose academic language is French. Simultaneous translation throughout the meeting of all presentations ensured that both English and the French speakers benefitted from the meeting proceedings. One of the outcomes of the meeting was the creation of the Senegalese Society of Human Genetics (S2HG), which supports one aim of the AfSHG, namely to create and sustain a network of African Human Genetics Societies, centres and individuals that together provide training, research and clinical services across the continent [1–5]. In the spirit of addressing the need for capacity development in the broad area of genomics, training workshops were held on research leadership, next-generation sequence data analysis in complex traits, and research grant writing.

## Background of the AfSHG

The AfSHG was founded in 2003 with the aim of equipping the African scientific community and policy-makers with information and practical knowledge to contribute to the field of genomics research and to attract global attention to the efforts of African scientists. A major objective of the society is to provide a forum for scientists in the broad enterprise of human genetics and genomics in Africa to meet, interact, network and collaborate. By achieving these goals, members of the AfSHG hope to bring attention to and help to facilitate the development of solutions to the huge public health burden of many rare and common diseases across the continent. It is indeed gratifying that the AfSHG and its members have successfully addressed some challenges while pushing forward with new strategies to address others [6–8]. Since its inaugural meeting, in Accra (Ghana) with the theme ‘*Biomedical Research in Africa with Emphasis on Genetics*’, the AfSHG is providing opportunities for networking and collaboration among professionals working on genetic and genomic issues relevant to Africa [6]. A list of its meetings to date is presented in Table 1. By continuing and sustaining its efforts, the AfSHG will help diminish the widening gap between Africa and the Western World in biomedical science. Furthermore, its collaboration with the H3Africa Consortium will help to foster a contemporary research approach to the study of genomics and environmental determinants of common diseases with the goal of improving the health of African populations [6–8]. The theme of the 9th conference was ‘*Strengthening Human Genetics Research in Africa*’ and is central to the mission of the AfSHG. In the absence of extensive research on the genetic contribution to diseases in diverse African populations, it would not be feasible to develop appropriate genetics services or to lay the ground work for the implementation of precision medicine approaches on the continent. Many African countries do not have Departments for Human Genetics as a stand-alone discipline. Therefore, we need to be creative in nesting our discipline and activities in existing structures in our tertiary institutions, hospitals and health care sectors, and to promote its contribution to patient care. Despite the limited resources available in Africa, the AfSHG is growing and working towards achieving its goals (http://www.afshg.org).

**Table 1.** AfSHG meetings since its inception in 2003

Meeting	Year	Venue	Theme
1	2003	Accra, Ghana	Biomedical Research in Africa with Emphasis on Genetics
2	2004	Washington, DC, USA	Sustaining the African Society of Human Genetics
3	2005	Johannesburg, South Africa	Human Genetic Variation in Africa
4	2006	Addis Ababa, Ethiopia	Human Genetic Variation and Disease
5	2007	Cairo, Egypt	Genomics Research in Africa: Implications for Disease Diagnosis, Treatment and Drug Development
6	2009	Yaunde, Cameroon	Human Origin, Genetic Diversity and Health [8]
7	2011	Cape Town, South Africa	Building Capacity for Genomic and Translational Research in Africa
8	2013	Accra, Ghana	Advancing Genomic Research in Africa: A Joint Conference of the AfSHG and H3Africa Consortium
9	2016	Dakar, Senegal	Strengthening Human Genetics Research in Africa

## Scientific highlights from the meeting

### Cancer genetics and genomic medicine

This session covered a broad range of haematological, cervical, colorectal and breast cancers. Two presentations highlighted the mechanism of nuclear remodelling and genomic instability in cancer and, more specifically, genomics of cervical cancer. Selected communications on haematological cancers commented on the usefulness of the qualitative in vitro diagnostic microarray in AML and genetic variants of *CYP2B6*, *GSTM1* and *GSTT1* that predict therapeutic response in patients treated with Imatinib. The importance of lymphocyte infiltrates in the outcome of colorectal tumours and the involvement of p53 variants in genetic predisposition to breast cancer were also discussed. The genomic medicine session included a particular interest in molecular diagnosis in clinical laboratories of albinism and of neurogenetic diseases, the latter focusing on families in Mali. Several communications highlighted results from target resequencing of genes involved in Parkinson's disease and novel variants in *SPG57*, *SPG10*, *SPG11* and *SPG42* in hereditary spastic paraplegia. There were also reports on genome-wide association studies in inflammatory bowel disease and body mass index variation.

### Medical genetics

Although there are few medical genetics and genetic counselling services and training programs in African countries, the number is growing and existing services are being strengthened. Several programs and services partner with non-African institutions, which strengthen their capacity for diagnostic testing and counselling of affected individuals and families. This session reported that services in Africa, specifically in Benin, Democratic Republic of Congo, Mali, Rwanda, Senegal, South Africa and Sudan, range from basic genetics, including cytogenetics and monogenic disease mutation testing, to high-level genetic tools such as next generation sequencing. There are, however, still huge disparities in services and access to services between African countries. By pulling together in a collaborative way, the existing platforms and expertise across the continent could be harnessed to have greater impact in the field. There is already evidence of shared training in Medical Genetics, where practitioners from Sudan and Cameroon are training at the University of Cape Town with the intention of returning to their respective institutions to establish medical genetic services.

### Genetics of infectious diseases

Africa has a high burden of infectious diseases, particularly malaria, HIV and tuberculosis. It is important to understand host susceptibility and also host pathogen interactions that drive the devastating epidemics. The meeting included presentations on malaria genomics, pharmacogenomics of antiretroviral therapy in HIV-infected populations and susceptibility to tuberculosis. These presentations highlighted the importance, relevance and timelines for ongoing research across the African continent in the context of emerging disease outbreaks as well as the increasing burden on health systems due to the rise of multi-morbidities (infectious and non-communicable) and complications arising from conditions that are now considered chronic diseases, such as HIV/AIDS. The changing epidemiology of infectious diseases in Africa and the importance of using genetics tools to tackle associated challenges were emphasised, with specific attention to the role of human host and pathogen genetic diversity in Africa.

### Human genetic diversity

A meeting in Africa would be remiss in not focusing on African and African descent population genetic diversity and its impact on health and susceptibility to disease. Several presentations highlighted allele frequency differences between African and non-African populations, but also across African populations, with potentially important implications for disease associations and the use of linkage disequilibrium mapping approaches to genome-wide association studies. The first African government-funded genome project, The Southern African Human Genome Programme, presented its pilot project demonstrating novel variant discovery in African populations using deep whole-genome sequencing data, and underscored the need to explore unique patterns of genetic diversity on the continent. In applying a public precision medicine approach, it is important to understand the spectrum of genetic variation across African populations.

### Ethical issues in genomic research

A major challenge for members of the AfSHG is the ethical issues surrounding voluntary participation and broad informed consent for genetic research conducted in African institutions [6, 7]. Two presenters highlighted the fact that there are no words for many genetics terms in African languages, and there is a great need to work together with policy-makers, the media and the public to help reduce fears and concerns surrounding genetics research. Another key issue is the interpretation of genetic sequencing data and novel variants in African populations. The paucity of data from African patients and populations makes it difficult to assign functional impact to genetic variation, and this is an increasing challenge as next-generation sequencing technologies, including exome and whole-genome sequencing, become more widely used. Joint AfSHG and H3Africa discussion sessions debated mechanisms for developing ethically and culturally acceptable designs of genetic research among Africans and how to deliver intensive training in genomic research.

### The young researcher forum

On Saturday, May 14, the Young Researcher Forum took place, aiming to provide young researchers with an opportunity to present their work and to learn from one another's experiences. The meeting had 150 participants, 24 orals 30 poster presentations. The keynote address was titled *Cancer in Africa*, *challenges and perspectives* with emphasis on collaborative research between Africa and the rest of the world. The presentations were on diverse themes in genetics and prizes were awarded for the best presenters.

## African societies of human genetics

**Fig. 1.**
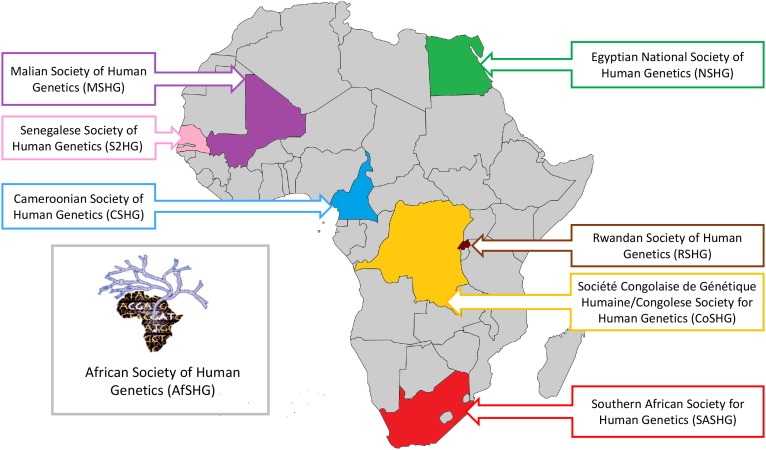
Current country-specific Societies of Human Genetics.

The number of country-specific Societies of Human Genetics across Africa is increasing and it is one of the objectives of the AfSHG to encourage and support such initiatives. This meeting brought together members from country-specific societies from Cameroon, Democratic Republic of Congo, Mali and Southern Africa (Fig. 1; Table 2 show all African Societies of Human Genetics). By hosting our meetings in different African countries, we encourage communities to formalise their associations and a major success of this meeting was the formal inauguration of the S2HG. The next AfSHG meeting will be held in Cairo in November 2017 under the auspices of The National Society of Human Genetics-Egypt.

**Table 2.** African country-specific societies of human genetics that form part of the African society of human genetics

Country	African Continent	Cameroon	Democratic Republic of Congo	Egypt	Mali	Rwanda	Senegal	South Africa
Name	African Society of Human Genetics	Cameroonian Society of Human Genetics	Société Congolaise de Génétique Humaine/Cong-olese Society for Human Genetics	Egyptian National Society of Human Genetics	Malian Society of Human Genetics	Rwandan Society of Human Genetics	Senegalese Society of Human Genetics	Southern African Society for Human Genetics
Abbreviation	AfSHG	CSHG	CoSHG	NSHG	MSHG	RSHG	S2GH	SASHG
Year formally established	2003	2009	2013	2005	2012	2017	2016	1986
Approximate number of members	950	80	30	49	102 registered	25 founder-members	50	350
Website	http://www.afshg.org			http://www.nshg-society.eg.net/			http://www.s2gh.org	http://sashg.org

## Towards sustainable human genetics research and services in Africa

The 9th AfSHG meeting demonstrated clearly that the existing language barriers between African investigators can be overcome; resulting in a highly interactive and productive conference. The Society will continue to foster collaboration between scientists and training the next generation of African researchers through its activities. The resulting insights into the genetic contributions to health and disease outcomes will facilitate the improvement of health across the continent.

## References

[1] de Vries J, The H3Africa policy framework: negotiating fairness in genomics. Trends in Genetics 2015; 31: 117–119. 25601285 10.1016/j.tig.2014.11.004 PMC4471134

[2] Mensah GA, H3Africa comes of age. Cardiovascular Journal of Africa 2015; 26(2 Suppl. 1): S3–S5. PMC4547554 25962946

[3] Ramsay M. Growing genomic research on the African continent: the H3Africa Consortium. South African Medical Journal 2015; 105: 1016–1017. 26792157 10.7196/SAMJ.2015.v105i12.10281

[4] Ramsay M, Sankoh O. African partnerships through the H3Africa Consortium bring a genomic dimension to longitudinal population studies on the continent. International Journal of Epidemiology 2016; 45: 305–308. 26659658 10.1093/ije/dyv187 PMC5841636

[5] Wonkam A, Sickle cell disease and H3Africa: enhancing genomic research on cardiovascular diseases in African patients. Cardiovascular Journal of Africa 2015; 26(2 Suppl. 1): S50–S55. 25962948 10.5830/CVJA-2015-040 PMC4547555

[6] Rotimi CN. Inauguration of the African Society of Human Genetics. Nature Genetics 2004; 36: 544. 15167923 10.1038/ng0604-544

[7] Wonkam A, Inauguration of the Cameroonian Society of Human Genetics. Pan African Medical Journal 2009; 3: 8. 21532717 10.4314/pamj.v3i1.52447 PMC2984290

[8] Wonkam A, Capacity-building in human genetics for developing countries: initiatives and perspectives in sub-Saharan Africa. Public Health Genomics 2010; 13: 492–494. 21135570 10.1159/000294171

